# Differences in the Physical Properties of Plant-Based Meat Alternatives Containing Root Vegetables

**DOI:** 10.3390/foods13233746

**Published:** 2024-11-22

**Authors:** Si-Yun Kim, Dong-Han Lee, Jeong-Jae Lee, Seo-Young Park, Seong-Gyu Choi, You-Jin Choi, Jung-Hyun Lee

**Affiliations:** 1 Department of Biosystems Engineering, Chungbuk National University, Cheongju 28644, Republic of Korea; 2024286005@chungbuk.ac.kr (S.-Y.K.); leedhan0105@naver.com (D.-H.L.); ljj2287@naver.com (J.-J.L.); 2 Research Institute, SY Solution Inc., Cheongju 28114, Republic of Korea; syp@kktong.co.kr (S.-Y.P.); csg0306@kktong.co.kr (S.-G.C.); eva@kktong.co.kr (Y.-J.C.)

**Keywords:** meat alternative, moisture content, PBMA, texture profile analysis

## Abstract

We investigated the textural characteristics of plant-based meat alternatives based on root vegetables, including *Platycodon grandiflorum*, *Codonopsis lanceolata root*, *Gastrodia elata blume*, and *Panax ginseng*. The samples with root vegetables had significantly higher moisture contents than those without because of the water retention capacity of dietary fiber contained in root vegetables. Heating affects the structures and interactions of the plant-based proteins and other ingredients. Therefore, from before to after heating, the L* values generally decreased, and the a* and b* values increased. During cooking, the hardness, gumminess, and chewiness of the sample containing *Platycodon grandiflorum* increased the most, and cohesiveness tended to increase The cooking loss was the lowest in the samples without root vegetable additives because the addition of root vegetables caused a decrease in *Pleurotus eryngii* content. The addition of root vegetables in samples had a positive effect on texture and overall acceptability in the sensory evaluation. Overall, the sample containing *Platycodon grandiflorum* was the most changed in terms of its physical properties. This study is expected to provide physical properties and foundational data for the future growth of the alternative food industry.

## 1. Introduction

Concerns are increasing worldwide regarding the meat production methods used in traditional livestock farming. These concerns are closely related to social issues such as environmental pollution, resource depletion, high carbon emissions, ethical aspects, and low sustainability. As such, interest in alternative meats that can be used to replace traditional meat is growing as meat supply instability and consumer interest in health increase [1]. Accordingly, alternative meats are being studied as one of the important parts of the food technology field, and the size of the alternative meat market and its share of the global meat market are expected to substantially grow with the development of food technology [2,3]. Alternative meat from the perspective of protein supply includes plant-, cell-, and insect-based products [4,5], but plant-based meat alternatives (PBMAs) have long been the most studied; thus, the term alternative meat generally refers to plant-based meat alternatives. PBMAs are products that mimic the taste and texture of traditional meat through the use of plant-based ingredients such as peas, soy, and coconut oil [6,7]. PBMAs can have the taste and texture of meat, as well as provide the same nutrition as meat for consumers, especially for many flexitarians, vegetarians, and vegans who want to benefit from these products while being considerate of environmental and ethical issues. Textured vegetable protein (TVP) was invented in the 1960s and is a major raw material used for the production of PBMAs; TVP can have similar structural and textural properties to consumable meats [8,9]. The process of structuring plant-based proteins serves as the foundation for texture formation in the production of PBMAs using TVP. Thus, this process has been studied using various technologies and methods, such as shear cells, wet spinning, electrospinning, mixing with hydrocolloids, ice/freeze structuring, and extrusion, but the only practical technologies are shear cells and extrusion [9,10,11]. Extrusion technology was developed in the 1930s and is currently the most widely used option for the production of PBMAs using TVP. Extrusion technology is used to deform and shape into a desired shape through the application of heat and pressure to a food mixture using single or twin screws to produce meat alternatives such as hamburger patties, hot dogs, bacon, lunch meat, and ground beef [10,11,12]. Consumer preference for PBMAs is still low despite advances in production technology. Global PBMA sales exceeded USD 10 billion in 2018 with continuous increases in demand and supply. Nevertheless, the dollar market share of PBMA based on the estimation of retail scanner data was less than 1% in all meat sales [13,14]. The numbers of PBMA consumers are low due to complex factors such as ethical values, eating habits, education level, sex, similarity to meat, taste, price, and convenience [15]. TVP and extrusion technologies have enabled the production of PBMAs that are similar to conventional meat in terms of taste, appearance, aroma, and nutritional properties [16], but PBMAs still differ in texture and taste from conventional meat. Vegetarians and occasional meat eaters do not expect PBMAs to be similar in texture and taste to meat [15]. However, for the growth and development of the PBMA industry, general consumers who want a product that is highly similar to meat must be considered. Therefore, researchers are actively developing products with properties similar to those of conventional meat by improving the properties of PBMAs. Mimicking the organized texture and water-binding ability of meat is a notable challenge facing improvements in the sensory properties of PBMAs [11,16]. The textural characteristics of PBMAs, such as hardness, chewiness, and water-binding ability, must be analyzed to identify differences according to the manufacturing process of PBMAs, considering factors that can be altered to increase the similarity of PBMAs to conventional meat. The differences in the characteristics of mixtures of various agricultural products must be considered to improve the texture and flavor of PBMAs. In particular, the inclusion of root vegetables such as *Platycodon grandiflorum*, *Codonopsis lanceolata root*, *Gastrodia elata blume*, and *Panax ginseng* could be used to change the flavor and physical properties of PBMAs due to their unique flavors, components, and viscosity. However, comparative analyses of the properties of PBMAs containing root vegetables have rarely been conducted. In this study, we considered the changes in the physical properties that occur in PBMA products by comparing those incorporating different root vegetables, including *Platycodon grandiflorum*, *Codonopsis lanceolata root*, *Gastrodia elata blume*, and *Panax ginseng.* We also considered their appropriate manufacturing process. Thus, our aim was to provide basic data for improving the flavor and texture of PBMAs and to evaluate the possibility of developing PBMA products using root vegetables.

## 2. Materials and Methods

### 2.1. Samples

The PBMA samples were manufactured with different types and contents of root vegetables using soy protein-based PVT by SY Solution Co., Ltd. (Cheongju-si, Republic of Korea). The root vegetables used included *Platycodon grandiflorum*, *Codonopsis lanceolata root*, *Gastrodia elata blume*, and *Panax ginseng*, which were produced in the Chungcheongbuk-do region of Korea and purchased at a local market. The composition of the PBMA samples is shown in Table 1. The control was a PBMA that did not contain root vegetables; RV-1 (root vegetable), RV-3, and RV-5 indicate samples with root vegetable contents of 1, 3, and 5%, respectively. Therefore, the number of treatments was 13 in total, based on 4 types of root vegetables, 3 types of contents, and 1 control treatment (0% root vegetables). The contents of root vegetables were determined at 1, 3, and 5% because, in preliminary experiments, structural damage occurred in a sample with root vegetable contents exceeding 5%. Additionally, the PVT and *Pleurotus eryngii* contents were adjusted according to the root vegetable content. Compound seasoning included modified tapioca starch, garlic powder, sugar, and starch. Samples were produced into a patty shape with a diameter of 100 mm using high-wet extrusion processing with a specific mixing ratio. The produced samples were cooled at room temperature for 1 h immediately after processing, frozen at −10 °C, and used within 1 week. In the experiment, the frozen samples were thawed in a refrigerator at 4 °C for 20 h and then analyzed.

The color and texture before and after heating, cooking loss, and shrinkage were compared to examine the changes in the physical properties of those samples that occurred with cooking. The samples were cooked in a preheated oven (RCD-300W, Rinnai, Nagoya, Japan) at 180 °C until the center temperature reached 75 °C and then cooled at room temperature for 30 min.

### 2.2. Moisture Content

The moisture contents before heating were measured following the AOAC standard [17]. We randomly obtained 10 g samples from 3 points in the patties, which were then dried in an experimental oven (OF3-15, JEIO TECH, Daejeon, Republic of Korea) at 105 °C for 24 h. The moisture content was determined from the ratio of the weight changes before and after drying.

### 2.3. Chromaticity

The color characteristics were assessed using a portable colorimeter (Cd-2500d, Konica Minolta, Tokyo, Japan) to determine the L (lightness), a* (redness; red–green), and b* (yellowness; yellow–blue) values. The L, a*, and b* values of the standard plate were 98.01, 0.08, and 0.06, respectively [18]. Each color value was measured at 15 random points on the sample surface and expressed as the average value. The total color difference (Δ*E*) was calculated using Equation (1) [19]:

(1) $$\Delta E=\sqrt{\Delta L^{2}+\Delta a^{2}+\Delta b^{2}}$$

where Δ*E* is the total color difference, while ΔL, Δa, and Δb are the difference in the L*, a*, and b* values between the control and each sample, respectively.

### 2.4. Texture Analysis

The texture of the samples was evaluated using a universal testing machine (UTM, EZ-SX 500N, SHIMADZU, Kyoto, Japan) to measure hardness, cohesiveness, gumminess, springiness, and chewiness. A cylindrical probe with a diameter of 25 mm was used, and the pretest, post-test, and test speeds were all set to 2.0 mm/s. Measurements were repeated 20 times by using twenty 10 mm cubes cut from each sample. And the texture value was expressed as the average value. The textural properties including cohesiveness, gumminess, springiness, and chewiness were calculated using Equations (2), (3), (4), and (5), respectively [20]:

(2) $$\mathrm{Cohesiveness}\;\left(\%\right)=\frac{D_{2}}{D_{1}}\times 100$$

$D_{1}$ and $D_{2}$ are the distances of the first and second occurring maximum stresses, respectively.

(3) $$\mathrm{Gumminess}\;\left(\%\right)=\mathrm{Hardness}\;\left(N\right)\times \mathrm{Cohesiveness}\;\left(\%\right)$$

(4) $$\mathrm{Springiness}\;\left(\%\right)=\frac{A_{2}}{A_{1}}\times 100$$

$A_{1}$ and $A_{2}$ are the areas of the first and second occurring maximum stresses, respectively.

(5) $$\mathrm{Chewiness}\;\left(g\right)=\frac{\mathrm{Springiness}}{100}\times \frac{\mathrm{Cohesiveness}}{100}\times \mathrm{Maximum}\;\mathrm{stress}$$

### 2.5. Cooking Loss

The cooking loss was determined from the ratio of the weight before and after heating. The weights were measured using an electronic balance (GF-4002A, AND, Tokyo, Japan). Cooking loss was calculated using Equation (6) [21]:

(6) $$\mathrm{Cooking}\;\mathrm{Loss}\;\left(\%\right)=\frac{\mathrm{Initial}\;\mathrm{Weight}-\mathrm{Weight}\;\mathrm{after}\;\mathrm{heating}}{\mathrm{Initial}\;\mathrm{Weight}}\times 100$$

### 2.6. Shrinkage

Shrinkage was determined from the calculated volume changes using the diameter and thickness of the sample before and after heating. The diameter and thickness were recorded at three different points using Vernier calipers (CD-20AX, Mitutoyo, Kanagawa, Japan). Samples were assumed to be cylindrical for volume calculations. Shrinkage was calculated using Equation (7) [21]:

(7) $$\mathrm{Shrinkage}\;\left(\%\right)=\frac{\mathrm{Initial}\;\mathrm{Volume}-\mathrm{Volume}\;\mathrm{after}\;\mathrm{heating}}{\mathrm{Initial}\;\mathrm{Volume}}\times 100$$

### 2.7. Sensory Evaluation

The sensory evaluation was measured by modifying Ryu et al.’s method [22]. Fifteen trained panelists (male and female) aged between 20 and 50 years old assessed the sensory characteristics before and after cooking samples. The panels were trained on sufficient knowledge, terminology, and evaluation criteria about the evaluation contents and samples. The samples with root vegetable contents of 5% were used, because the differences in physical characteristics by contents of root vegetables were low in other measurements. The sensory evaluation used a 9-point hedonic scale (1 = extremely bad or undesirable, 9 = extremely good or desirable). The panels evaluated appearance, aroma, flavor, texture, and overall acceptability.

### 2.8. Statistical Analysis

The data were analyzed using one-way analysis of variance, and the SPSS statistical package (IBM SPSS Statistics 27.0, IBM, Armonk, NY, USA) was used to analyze the data. Differences according to the different conditions were established using one-way ANOVA and the Tukey–Kramer multiple range test with a significance level of *p* < 0.5.

## 3. Results and Discussion

### 3.1. Moisture Content

The moisture content before heating was calculated on a wet basis, and the results are presented in Table 2. The moisture contents of the samples containing root vegetables were significantly higher than those of the controls. This result aligns with the findings of Joo and Choi [23], who found that higher vegetable contents in alternative meat patties increased the dietary fiber’s ability to retain moisture, thereby reducing water loss and increasing the moisture contents of the patties. However, the moisture contents of the patties containing different root vegetable types and contents did not significantly differ.

### 3.2. Chromaticity

The addition of root vegetables significantly affected the color of the PBMAs. The color values and appearances of the samples before and after heating for the different root vegetable types and contents are shown in Table 3 and Table 4, respectively. The lightness (L*) and yellowness (b*) values of the control were higher than those of the samples containing root vegetables, except for the b* value in the *Platycodon grandiflorum* samples. In previous studies, the color of PBMAs changed with the addition of plant-based materials such as *Tremella fuciformis* [24], *Pleurotus eryngii* [25], and *Morus alba* L. leaves [26]. Decreases in the L* values were mainly observed. Additionally, the color values tended to increase as the root vegetable content increased, but the difference was small or not significant. This trend was more pronounced in the samples after heating. From before to after heating, the L* values generally decreased, and the a* and b* values increased. The total color difference (ΔE) between the control and other samples before heating averaged 7.25, but the average ΔE after heating increased to 9.60, whereas the changes in the other color values were not significant for the different root vegetable types and contents. Heating promotes changes in the structures and interactions between the proteins and other ingredients [23]. Therefore, the colors of the samples changed with heating, which was due to the main ingredients, such as PVT and *Pleurotus eryngii*; on the other hand, the root vegetables had a minor effect on color, so it was considered that the significant difference between the control and samples was reduced. Table 4 shows that the difference in appearance between the control and the samples containing root vegetables or between before and after heating could be distinguished with the naked eye. However, these differences were not apparent with the naked eye among the samples with different root vegetable types and contents.

### 3.3. Texture Analysis

The addition of root vegetables such as *Platycodon grandiflorum* and *Panax ginseng* was shown to affect the texture of PBMA. The texture values of samples before and after heating are shown in Table 5 and Table 6, respectively. As shown in Table 5, samples with added *Platycodon grandiflorum* or *Panax ginseng* showed a decrease in the overall texture value compared to the control. Samples with added *Codonopsis lanceolata root* or *Gastrodia elata blume* generally showed no significant difference from the control. Also, no significant differences were found depending on root vegetable content. The difference in texture value between the control and samples with added root vegetables was reduced by heating the samples. As shown in Table 6, the hardness, gumminess, and chewiness of all samples after heating significantly increased by more than three times. In particular, the sample with added *Platycodon grandiflorum* showed the largest increase and the highest values, with the value increasing by about seven times. Although the texture values after heating significantly changed compared to before heating, the difference in texture value between the control and samples with added root vegetables showed no significant difference, except for a sample with added *Platycodon grandiflorum*. This result was considered to occur due to the increased influence of the main components of the sample such as PVT and *Pleurotus eryngii* compared to root vegetables, as in the case of chromaticity. In previous studies, the textures of PBMA were affected by the addition of materials such as *Morus alba* L. leaf [26], red yeast rice [27], and *Haematococcus pluvialis* [28]. Although results may vary depending on experimental conditions, the effect on texture differed depending on the food additives. In case of hardness, PBMA with added *Platycodon grandiflorum*, red yeast rice [27], or *Haematococcus pluvialis* [28] decreased, while PBMA with added *Morus alba* L. leaf [26] increased. The decrease in hardness may be a good alternative for consumers who want a softer texture. In general, plant-based patties had lower hardness than animal-based patties due to the biological force of muscle tissue in patty with meat [27]. Thus, the addition of *Platycodon grandiflorum* to PBMA may be helpful for the production of patties with a texture similar to that of conventional meat.

### 3.4. Cooking Loss and Shrinkage

Less cooking loss occurred in the controls than in the samples containing root vegetables. The cooking loss of the samples is shown in Table 7, which was higher by 2.12 to 9.91% compared with that of the control. The cooking loss of the sample containing *Platycodon grandiflorum* was the largest. Cooking loss is caused by the evaporation and leakage of the moisture in a sample. Samples with higher moisture contents may lose less moisture even though their cooking loss is larger. However, the measured cooking loss values indicated that the controls lost less moisture, even though the moisture contents of the samples containing root vegetables were higher, as shown in Table 2. In previous studies, the cooking loss and shrinkage of animal-based patties were higher owing to the cooking process compared with those of plant-based patties, which could be due to the dietary fiber incorporated during the initial formulation [23,27]. Notably, *Pleurotus eryngii* is rich in dietary fiber [29]. The higher cooking loss of the samples containing root vegetables may have been affected by the decrease in *Pleurotus eryngii* content (i.e., the decrease in dietary fiber content) during formulation. Cooking loss also increased with increasing root vegetable content. However, shrinkage did not significantly differ based on increasing cooking loss. This may have occurred because the structures of the samples had already denatured and aggregated during the production process [23], so the effect of the root vegetable content was weaker.

### 3.5. Sensory Evaluation

The results of sensory evaluation are presented in Table 8. In all evaluation categories, samples with added root vegetables received higher scores than the control, with a particularly significant increase in the texture and overall acceptability score, confirming the positive impact of root vegetable addition on texture and overall acceptability. The flavor score more significantly increased samples with added *platycodon grandiflorum* and *codonopsis lanceolata root* than the control. Scores in all categories were generally higher after cooking compared to before cooking. Overall, the sensory evaluation values of the sample with added *platycodon grandiflorum* showed higher scores than other samples, including the control.

## 4. Conclusions

We investigated the changes that occurred in the physical properties of PBMAs by adding root vegetables including *Platycodon grandiflorum*, *Codonopsis lanceolata root*, *Gastrodia elata blume*, and *Panax ginseng*. The addition of root vegetables significantly changed the physical properties of the PBMAs. The chromaticity and texture tended to be lower, whereas moisture content and cooking loss were higher in the samples containing root vegetables compared with the control. Sensory evaluation values for flavor, texture, and overall acceptability were improved. In particular, the sample containing *Platycodon grandiflorum* was the most changed by its addition. In the overall measurements, no significant differences among the different contents of root vegetables were found. The effect of their addition was weak because the root vegetable contents were low. However, increasing the root vegetable content may be undesirable. An increase in certain components can lead to a decrease in other components, which may decrease the quality of PBMAs, manifested as increased molding difficulty, color changes, and structural damage. The addition of root vegetables in contents exceeding 5% led to structural damage during the PBMA production process.

The sophisticated combination of plant proteins, various food additives, and production technologies is important for enhancing the flavor and appearance of PBMAs, particularly when overall quality enhancement is achieved through ingredient synergy [2]. Thus, analyzing the changes in the characteristics of the mixtures of various agricultural products is crucial. Our findings suggest the potential for using root vegetables to increase the quality of PBMA products. The addition of *Platycodon grandiflorum* to PBMAs may affect the quality of the final product, such as its moisture content, texture, flavor, and cooking loss. Further research is warranted to explore different blending ratios of root vegetables and the effects of the inclusion of other types of root vegetables on plant-based meat alternatives, including the analysis of chemical change and nutritional value.

## Figures and Tables

**Table 1:** The components of PBMAs containing root vegetables.

	Control (%)	RV-1 (%)	RV-3 (%)	RV-5 (%)
Purified water	32	32	32	32
PVT	31	31	30	30
*Pleurotus eryngii*	15	14	14	12
Rape seed oil	16	16	16	16
Root vegetable	0	1	3	5
Compound seasoning	6	6	5	5

**Table 2:** The moisture contents of the samples before heating according to root vegetable content. All values are presented as mean ± SD (*n* = 3). Significant differences (*p* < 0.05) between different root vegetable types and contents are indicated with lowercase letters (a, b).

Sample		Moisture Contents (% w.b.)
Control		55.53 ± 0.31 ^a^
*Platycodon grandiflorum*	RV-1	60.93 ± 0.62 ^b^
	RV-3	61.30 ± 0.94 ^b^
	RV-5	61.07 ± 0.09 ^b^
*Codonopsis lanceolata root*	RV-1	60.30 ± 0.24 ^b^
	RV-3	60.23 ± 0.34 ^b^
	RV-5	61.15 ± 0.97 ^b^
*Gastrodia elata blume*	RV-1	60.87 ± 0.81 ^b^
	RV-3	61.13 ± 0.45 ^b^
	RV-5	61.80 ± 1.02 ^b^
*Panax ginseng*	RV-1	59.67 ± 0.46 ^b^
	RV-3	60.90 ± 0.49 ^b^
	RV-5	61.20 ± 0.93 ^b^

**Table 3:** The chromaticity of the samples before and after heating. All values are presented as the mean ± SD (*n* = 15). Significant differences (*p* < 0.05) between the different root vegetable types and contents are labeled by lowercase letters (a–g).

Sample		Before Heating			After Heating		
		L*	a*	b*	L*	a*	b*
control		71.95 ± 1.17 ^a^	3.60 ± 0.45 ^efg^	14.75 ± 0.79 ^c^	62.50 ± 2.22 ^a^	5.10 ± 0.82 ^a^	17.98 ± 1.36 ^a^
*Platycodon grandiflorum*	RV-1	66.32 ± 3.25 ^e^	5.47 ± 0.77 ^bc^	17.17 ± 1.04 ^b^	54.18 ± 2.86 ^bc^	4.48 ± 0.96 ^a^	12.61 ± 1.84 ^c^
	RV-3	69.03 ± 2.40 ^b^	5.91 ± 0.43 ^b^	18.33 ± 0.58 ^b^	52.08 ± 2.70 ^c^	5.03 ± 0.76 ^a^	14.48 ± 1.71 ^b^
	RV-5	67.30 ± 2.95 ^bc^	6.78 ± 1.22 ^a^	20.65 ± 1.66 ^a^	52.05 ± 2.79 ^c^	5.30 ± 0.80 ^a^	14.36 ± 1.27 ^b^
*Codonopsis* *lanceolata root*	RV-1	56.59 ± 3.68 ^bc^	4.87 ± 0.22 ^cd^	13.33 ± 0.95 ^d^	55.07 ± 2.99 ^bc^	4.88 ± 0.74 ^a^	14.00 ± 0.88 ^b^
	RV-3	63.09 ± 1.37 ^d^	4.30 ± 0.46 ^de^	13.32 ± 1.15 ^d^	53.02 ± 2.65 ^bc^	5.17 ± 0.86 ^a^	14.23 ± 1.35 ^b^
	RV-5	63.03 ± 2.89 ^d^	4.24 ± 0.72 ^de^	13.20 ± 0.81 ^d^	53.14 ± 3.54 ^bc^	4.30 ± 0.91 ^a^	14.32 ± 1.38 ^b^
*Gastrodia elata blume*	RV-1	68.22 ± 3.06 ^b^	3.36 ± 0.96 ^fg^	12.17 ± 2.24 ^de^	53.97 ± 3.20 ^bc^	4.74 ± 1.11 ^a^	14.25 ± 1.35 ^b^
	RV-3	67.43 ± 1.71 ^bc^	3.71 ± 0.50 ^efg^	11.71 ± 0.83 ^e^	55.63 ± 1.83 ^bc^	4.56 ± 0.70 ^a^	14.40 ± 1.25 ^b^
	RV-5	67.12 ± 2.09 ^bc^	3.21 ± 0.29 ^fg^	11.21 ± 0.74 ^e^	52.55 ± 2.99 ^bc^	5.23 ± 1.15 ^a^	14.24 ± 1.42 ^b^
*Panax* *ginseng*	RV-1	66.89 ± 0.88 ^bc^	3.87 ± 0.58 ^ef^	12.59 ± 1.19 ^de^	56.01 ± 3.51 ^b^	4.50 ± 0.97 ^a^	14.28 ± 1.32 ^b^
	RV-3	64.98 ± 2.21 ^cd^	3.87 ± 0.54 ^ef^	12.56 ± 0.88 ^de^	53.62 ± 2.72 ^bc^	5.34 ± 0.69 ^a^	14.50 ± 0.74 ^b^
	RV-5	67.84 ± 1.47 ^bc^	3.06 ± 0.51 ^g^	12.17 ± 1.51 ^de^	53.35 ± 3.00 ^bc^	5.17 ± 0.85 ^a^	14.71 ± 1.05 ^b^

**Table 4:** The appearances of samples before and after heating.

Control	Before Heating			After Heating		
	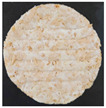			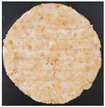		
	RV-1	RV-3	RV-5	RV-1	RV-3	RV-5
*Platycodon grandiflorum*	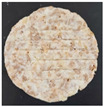	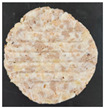	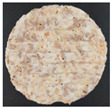	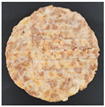	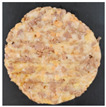	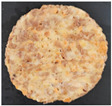
*Codonopsis* *lanceolata root*	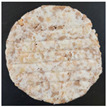	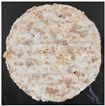	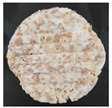	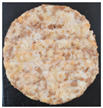	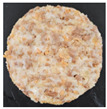	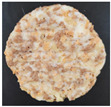
*Gastrodia elata blume*	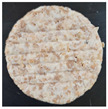	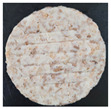	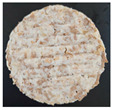	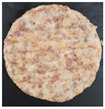	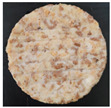	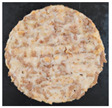
*Panax* *ginseng*	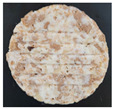	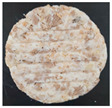	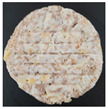	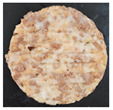	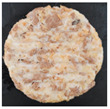	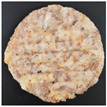

**Table 5:** Texture profile analysis of meat alternatives by root vegetable content before heating. All values are mean ± SD (*n* = 20). Significant differences (*p* < 0.05) between the samples with different root vegetable types and contents are labeled with lowercase letters (a–e).

		Hardness (N)	Cohesiveness (%)	Gumminess (%)	Springiness (%)	Chewiness (g)
Control		1.65 ± 0.37 ^ab^	59.66 ± 4.04 ^ab^	98.08 ± 20.06 ^ab^	69.56 ± 5.45 ^a^	0.68 ± 0.14 ^abc^
*Platycodon grandiflorum*	RV-1	1.05 ± 0.34 ^d^	50.04 ± 6.86 ^c^	52.30 ± 18.61 ^e^	56.83 ± 7.67 ^b^	0.31 ± 0.14 ^d^
	RV-3	1.07 ± 0.34 ^d^	49.75 ± 5.93 ^c^	52.54 ± 15.36 ^e^	54.97 ± 5.34 ^b^	0.29 ± 0.10 ^d^
	RV-5	1.02 ± 0.41 ^d^	50.33 ± 9.10 ^c^	49.26 ± 14.34 ^e^	57.05 ± 9.40 ^b^	0.28 ± 0.11 ^d^
*Codonopsis lanceolata root*	RV-1	1.67 ± 0.37 ^ab^	62.48 ± 3.68 ^ab^	103.85 ± 20.99 ^ab^	72.22 ± 4.06 ^a^	0.75 ± 0.16 ^ab^
	RV-3	1.47 ± 0.27 ^bc^	58.63 ± 6.94 ^b^	86.41 ± 19.30 ^bcd^	70.43 ± 5.85 ^a^	0.61 ± 0.17 ^bc^
	RV-5	1.93 ± 0.64 ^a^	58.83 ± 3.50 ^b^	112.49 ± 34.20 ^a^	72.61 ± 4.05 ^a^	0.83 ± 0.28 ^a^
*Gastrodia elata blume*	RV-1	1.56 ± 0.34 ^abc^	64.42 ± 4.17 ^ab^	99.93 ± 19.76 ^ab^	74.58 ± 3.82 ^a^	0.74 ± 0.14 ^ab^
	RV-3	1.59 ± 0.29 ^abc^	59.09 ± 4.59 ^b^	93.40 ± 14.63 ^abc^	70.71 ± 3.97 ^a^	0.66 ± 0.12 ^abc^
	RV-5	1.72 ± 0.49 ^ab^	60.64 ± 4.53 ^ab^	104.16 ± 29.83 ^ab^	73.48 ± 5.55 ^a^	0.77 ± 0.25 ^ab^
*Panax* *ginseng*	RV-1	1.24 ± 0.39 ^cd^	62.22 ± 5.24 ^ab^	75.77 ± 21.08 ^cd^	70.40 ± 5.64 ^a^	0.53 ± 0.15 ^c^
	RV-3	1.04 ± 0.40 ^d^	65.08 ± 6.36 ^a^	67.95 ± 27.78 ^de^	73.17 ± 6.00 ^a^	0.51 ± 0.24 ^c^
	RV-5	1.10 ± 0.36 ^d^	62.42 ± 6.43 ^ab^	68.36 ± 21.55 ^de^	73.95 ± 6.01 ^a^	0.51 ± 0.17 ^c^

**Table 6:** The results of the texture profile analysis of meat alternatives with different root vegetable contents after heating. All values are presented as the mean ± SD (n = 20). Significant differences (p < 0.05) between the root vegetable types and contents are indicated with lowercase letters (a–e).

Sample		Hardness (N)	Cohesiveness (%)	Gumminess (%)	Springiness (%)	Chewiness (g)
Control		5.13 ± 1.62 ^c^	56.28 ± 4.04 ^abcd^	285.42 ± 85.70 ^bc^	72.98 ± 5.33 ^a^	2.08 ± 0.64 ^abc^
*Platycodon grandiflorum*	RV-1	7.06 ± 1.32 ^ab^	51.93 ± 6.86 ^de^	366.47 ± 71.59 ^ab^	71.74 ± 4.17 ^a^	2.64 ± 0.60 ^a^
	RV-3	7.52 ± 3.23 ^a^	53.03 ± 5.93 ^bcde^	383.88 ± 138.72 ^a^	71.28 ± 4.46 ^a^	2.71 ± 0.93 ^a^
	RV-5	6.77 ± 2.03 ^ab^	54.16 ± 9.10 ^abcde^	361.27 ± 93.12 ^ab^	71.88 ± 4.83 ^a^	2.59 ± 0.67 ^ab^
*Codonopsis lanceolata root*	RV-1	4.99 ± 1.24 ^c^	57.93 ± 3.68 ^abc^	287.13 ± 66.31 ^bc^	72.18 ± 5.22 ^a^	2.09 ± 0.56 ^abc^
	RV-3	4.95 ± 1.58 ^c^	59.14 ± 6.94 ^a^	287.64 ± 82.20 ^bc^	74.67 ± 3.10 ^a^	2.15 ± 0.63 ^abc^
	RV-5	5.14 ± 1.77 ^c^	58.26 ± 3.50 ^ab^	298.13 ± 112.01 ^bc^	72.18 ± 4.87 ^a^	2.17 ± 0.90 ^abc^
*Gastrodia elata blume*	RV-1	4.93 ± 1.20 ^c^	56.34 ± 4.17 ^abcd^	273.47 ± 51.55 ^c^	71.95 ± 3.94 ^a^	1.97 ± 0.38 ^bc^
	RV-3	4.99 ± 1.07 ^c^	52.49 ± 4.59 ^de^	262.12 ± 59.07 ^c^	71.51 ± 3.50 ^a^	1.88 ± 0.47 ^c^
	RV-5	4.55 ± 1.25 ^c^	52.02 ± 4.53 ^de^	235.71 ± 66.27 ^c^	72.67 ± 5.11 ^a^	1.72 ± 0.51 ^c^
*Panax* *ginseng*	RV-1	4.37 ± 0.94 ^c^	56.62 ± 5.24 ^abcd^	244.64 ± 46.52 ^c^	70.91 ± 2.87 ^a^	1.73 ± 0.34 ^c^
	RV-3	4.97 ± 1.82 ^c^	49.02 ± 6.36 ^e^	242.19 ± 89.34 ^c^	70.01 ± 4.58 ^a^	1.69 ± 0.61 ^c^
	RV-5	5.88 ± 1.31 ^bc^	52.78 ± 6.43 ^cde^	307.99 ± 59.26 ^abc^	74.17 ± 3.78 ^a^	2.29 ± 0.45 ^abc^

**Table 7:** The cooking loss and shrinkage of the samples that occurred with heating. All values are reported as the mean ± SD (*n* = 3). Significant differences (*p* < 0.05) between the root vegetable types and contents are indicated by lowercase letters (a–e).

Sample		Cooking Loss (%)	Shrinkage (%)
Control		13.43 ± 0.80 ^e^	17.27 ± 1.62 ^ab^
*Platycodon grandiflorum*	RV-1	20.06 ± 0.62 ^b^	19.60 ± 2.59 ^ab^
	RV-3	23.33 ± 0.89 ^a^	27.14 ± 6.60 ^a^
	RV-5	23.22 ± 0.90 ^a^	26.18 ± 2.84 ^a^
*Codonopsis lanceolata root*	RV-1	15.54 ± 0.55 ^d^	17.34 ± 2.88 ^ab^
	RV-3	17.11 ± 0.44 ^cd^	17.54 ± 1.56 ^ab^
	RV-5	16.22 ± 0.59 ^d^	16.50 ± 0.87 ^ab^
*Gastrodia elata blume*	RV-1	16.23 ± 0.57 ^d^	19.78 ± 3.97 ^ab^
	RV-3	17.24 ± 0.48 ^cd^	19.07 ± 4.49 ^ab^
	RV-5	16.09 ± 0.81 ^d^	15.03 ± 0.57 ^b^
*Panax ginseng*	RV-1	15.84 ± 0.97 ^d^	18.38 ± 1.39 ^ab^
	RV-3	16.34 ± 0.30 ^d^	20.04 ± 0.29 ^ab^
	RV-5	18.54 ± 0.56 ^bc^	22.04 ± 2.20 ^ab^

**Table 8:** The sensory evaluation of the samples with RV-5 and the control. All values are reported as the mean ± SD (*n* = 15). Significant differences (*p* < 0.05) between the root vegetable types and contents are indicated by lowercase letters (a–f).

Sample	Cooking	Sensory Evaluation (Point)				
		Appearance	Aroma	Flavor	Texture	Overall Acceptability
Control	Before	6.3 ± 0.6 ^a^	5.7 ± 0.4 ^a^	-	4.7 ± 0.3 ^a^	5.7 ± 0.4 ^a^
	After	6.8 ± 0.5 ^bcde^	6.2 ± 0.5 ^bc^	6.6 ± 0.4 ^a^	6.0 ± 0.5 ^b^	6.4 ± 0.4 ^c^
*Platycodon* *grandiflorum*	Before	6.7 ± 0.3 ^abcd^	5.9 ± 0.4 ^ab^	-	6.4 ± 0.3 ^c^	6.6 ± 0.2 ^cd^
	After	7.2 ± 0.3 ^e^	6.9 ± 0.2 ^d^	7.6 ± 0.4 ^b^	7.3 ± 0.3 ^d^	7.5 ± 0.2 ^f^
*Codonopsis* *lanceolata root*	Before	6.5 ± 0.3 ^abc^	6.0 ± 0.4 ^ab^	-	6.8 ± 0.3 ^c^	6.0 ± 0.2 ^ab^
	After	7.1 ± 0.2 ^e^	6.5 ± 0.2 ^cd^	7.2 ± 0.4 ^b^	7.4 ± 0.2 ^d^	7.1 ± 0.3 ^ef^
*Gastrodia* *elata blume*	Before	6.4 ± 0.3 ^ab^	5.8 ± 0.3 ^ab^	-	6.6 ± 0.3 ^c^	5.9 ± 0.3 ^ab^
	After	6.9 ± 0.2 ^cde^	6.5 ± 0.3 ^cd^	6.7 ± 0.2 ^a^	7.2 ± 0.2 ^d^	6.9 ± 0.3 ^de^
*Panax* *ginseng*	Before	6.5 ± 0.3 ^abc^	5.9 ± 0.4 ^ab^	-	6.7 ± 0.3 ^c^	6.3 ± 0.4 ^bc^
	After	7.0 ± 0.2 ^de^	6.8 ± 0.3 ^d^	6.8 ± 0.3 ^a^	7.3 ± 0.2 ^d^	7.3 ± 0.3 ^f^

(-): The flavors of the samples before cooking were not measured.

## Data Availability

The data presented in this study are available on request from the corresponding author due to privacy reasons.
